# Bioavailability of nanomaterials: bridging the gap between nanostructures and their bioactivity

**DOI:** 10.1093/nsr/nwac119

**Published:** 2022-06-17

**Authors:** Mingjing Cao, Chunying Chen

**Affiliations:** CAS Key Laboratory for Biomedical Effects of Nanomaterials and Nanosafety and CAS Center for Excellence in Nanoscience, National Center for Nanoscience and Technology of China, China; CAS Key Laboratory for Biomedical Effects of Nanomaterials and Nanosafety and CAS Center for Excellence in Nanoscience, National Center for Nanoscience and Technology of China, China; University of Chinese Academy of Sciences, China; The GBA National Institute for Nanotechnology Innovation, China

## Abstract

Instead of the nanostructure and biological activity, this perspective highlights the metabolic processes from biotransformation to bioavailability, to bridge the gap between the cradle (structure design) and endpoint (efficacy and/or safety) of nanomedicines.

Nanomaterials (NMs) have enormous potential as nanomedicines for diagnosing and fighting various diseases. Researchers make every endeavor to design diverse nanomedicines with novel nanostructures and smart payloads. But how stable are nanomedicines before and after exerting their functions at target sites? What can we do to maximize the efficiency of nanomedicines? These questions have concerned researchers over the past two decades. The *in vivo* metabolic processes of nanomaterials are a key factor in understanding the safety and efficacy (end point) of nanomedicines. Clarification of these processes may bridge the gap between the cradle (structure design) and end point (efficacy and/or safety) of nanomedicines. Four possible metabolic phenomena have been revealed so far for NMs [1,2]: their elimination through renal and hepatobiliary systems, their interception in the mononuclear phagocyte system (MPS), their biodegradation and utilization in the liver and, finally, their excretion from the body after a gradual breakdown.

Trace-element-based NMs (TENMs) are potentially bioactive species. The 21 trace elements (TEs) for nutrition, documented in a WHO report (*Trace elements in human nutrition and health*, 1996), such as Se, I, Mo, Fe, Cu, Zn, Cr, Co and Mn, play vital roles in maintaining the body’s metabolism and function. As an essential part of proteins, enzymes and their cofactors, these elements participate in important biochemical reactions. Here, we define the ‘bioavailability of NMs’ as a process in which metabolites of NMs play essential active roles in biomolecules, or regulate biological activities or efficacy as pathway modulators.

Figure 1A shows the protein corona-impacted transport–transformation–bioavailability process of TENMs. The TENMs@protein corona complexes formed first, followed by their transportation to target organs under the guidance of the protein corona. Nano-bio interactions between TENMs and various biochemical factors [3], such as enzymes, oxidants, reductants and acidity, induce the dissolution of TENMs. As engineered surface coatings and the protein corona are likely sensitive to enzymes present in biological environments, the TE-based nanocores may be degraded after the disassembly of their surface ligands. It is well known that the degradation is highly dependent on chemical compositions. pH, enzymes, reactive oxidative species (ROS) and reductants are major factors possibly involved in the decomposition of TENMs. Ultimately, metal ions, cofactors and biomolecules derived from biodegradable TENMs could become bioavailable by their incorporation into enzymes, and be involved in the synthesis of proteins directly or through acting as pathway regulators. Transformation and bioavailability are largely dictated by the biological microenvironment surrounding TENMs, which is in turn determined by their physicochemical properties (i.e. size, shape, surface charge and ligands). For example, large-sized (>150 nm), negatively charged and hydrophilic NMs are preferentially arrested by liver resident macrophages that are mainly responsible for the degradation of liver-targeting NMs. Furthermore, the protein corona adsorption affected by their intrinsic characteristics confers a new biological identity for TENMs and determines cellular uptake, biodistribution, transformation, biological activities and safety. Opsonins, such as complements and immunoglobulins, enriched in the protein corona, induce the sequestration of NMs in the MPS, while the adsorbed desoponins (e.g. albumin, apolipoproteins) reduce cellular uptake by immune cells. The serum protein corona also acts as an important modulator on the biotransformation of NMs by inducing sulfidation or accelerating the biodegradation rate. Therefore, precise structure design of TENMs and control of protein corona adsorption are critical for the delivery and utilization of trace elements.

**Figure 1.**
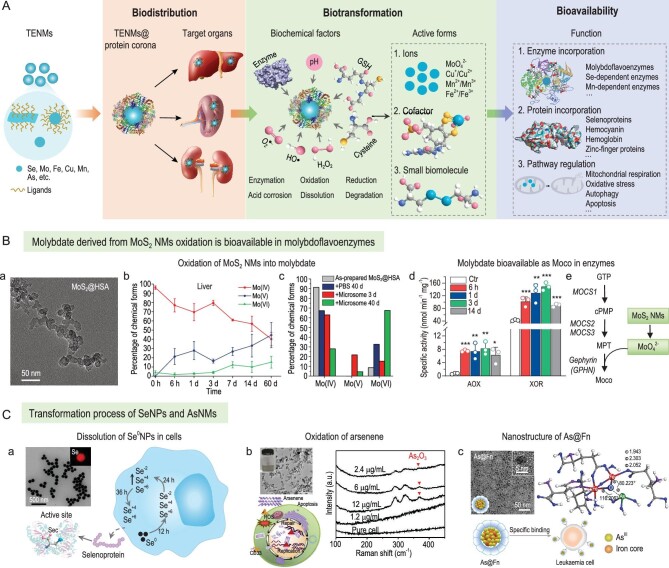
(A) Schematic for the bioavailability of TENMs *in vivo*. The process was initiated with the formation of a TENMs@protein corona, followed by the transport and transformation of NMs modulated by the protein corona. The oxidation/reduction/dissolution forms derived from TENMs ultimately become bioavailable by their incorporation into biomolecules as cofactors, involving proteins directly, or modulating biological activities as pathway regulators. (B) MoS_2_ NMs are bioavailable as molybdenum cofactors (Moco) in enzymes. (a) Transmission electron microscopy (TEM) image of MoS_2_@HSA nanocomplexes. (b) Molybdenum chemical forms and ratios in livers, calculated from measurements of molybdenum K-edge X-ray absorption near edge spectroscopy. (c) Oxidation of MoS_2_ by liver microsomes over time, determined by X-ray photoelectron spectroscopy. (d) Increased activities of AOX and XOR in mouse livers due to molybdate participating in the biosynthesis of Moco (e). Adapted with permission from ref. [4]. Copyright 2021 Springer Nature. (C) Biotransformation and bioavailability of SeNPs and arsenic-based NMs (AsNMs). (a) TEM image and dissolution process of SeNPs with incorporation in selenoproteins. Sec, selenocysteine. Adapted with permission from ref. [6]. Copyright 2020 Elsevier Ltd. (b) TEM image of arsenene, apoptosis of APL cells induced by arsenene and oxidation of arsenene in cells measured by Raman spectroscopy. Adapted with permission from ref. [7]. Copyright 2019 WILEY-VCH Verlag GmbH and Co. KGaA, Weinheim. (c) Nanostructure and target ability against diverse leukaemia cells of As@Fn nanocomplex. Adapted with permission from ref. [8]. Copyright 2021 Springer Nature.

So far, TE-based nanomedicines have been increasingly designed in the biomedical field due to their outstanding biocompatibility and utility. Figure 1B and C give three examples to illuminate the transformation and bioavailability of TENMs. The MoS_2_ nanosheet is a rising star of two-dimensional (2D) transition metal dichalcogenides, entailing excellent properties. We found that 2D MoS_2_ nanosheets modified with serum albumins underwent a biodistribution–biotransformation–bio-availability process bridged by the protein corona [4]. MoS_2_ NMs sequestrated in the liver and spleen were oxidized to molybdate by phase I enzymes and ROS (Fig. 1B). Meanwhile, activities of aldehyde oxidase (AOX) and xanthine oxidoreductase (XOR), two main molybdoflavoenzymes, were enhanced since molybdate participated in the biosynthesis of molybdenum cofactors and acted as active sites in molybdenum enzymes. The increased activities of AOX and XOR may induce beneficial or harmful consequences by generating active products, such as NO and ROS, or interacting with anti-cancer, anti-microbial and anti-viral drugs.

Similar to molybdenum-based NMs, selenium nanoparticles (SeNPs) could be utilized by incorporating active species into selenoproteins. Se^0^NPs coated with albumins had a metabolic pathway different to that of inorganic selenite [5]. Indeed, Se^0^NPs were mainly transformed into selenocystine, while selenite was more easily oxidized into selenate. In another work, bare Se^0^NPs and modified ones (PVP, PEG, etc.) were designed to boost cytokine-induced killer-cells-based cancer immunotherapy (Fig. 1C) [6], where Se^0^NPs were metabolized into selenocystine, an essential factor in the synthesis of multiple selenoproteins responsible for immune activation, including glutathione peroxidases and thioredoxin reductases.

Arsenic-based NMs (AsNMs) are effective for the treatment of acute promyelocytic leukaemia (APL) (Fig. 1C). Arsenene, a novel 2D nanosheet, was synthesized against APL cells by inducing cellular apoptosis [7]. Moreover, a As@Fn nanocomplex with superior anti-leukaemia efficacy was constructed by encapsulating H_3_AsO_3_ into a ferritin (Fn) cage with the form of a Fe-O-As complex [8]. Arsenene can be oxidized to As_2_O_3_ in APL cells [7]. AsNMs may also be metabolized to inorganic (arsenate, arsenite) and organic arsenics (monomethyl arsonate, dimethyl arsenate). The arsenic metabolites could become bioavailable via different signaling routes, for example by binding to the Zn finger of DNA repair enzymes and by activating nicotinamide adenine dinucleotide phosphate (NADPH) oxidase. Trivalent arsenic (As_2_O_3_) can induce APL cell apoptosis by triggering the degradation of PML-RARα, an abnormal protein resulting in immortality [9]. The enhanced ability against APL of AsNMs may be contributed by the biological functions of the metabolites.

It is also possible for other TENMs (Mn, Fe, Cu, Zn, etc.) to become bioavailable and involved in metabolism and physiological activities. For example, Mn-based NMs engineered as nanovaccines [10] have the potential to release Mn^2+^ to further activate the cyclic GMP–AMP synthase (cGAS)-stimulator of interferon genes (STING) pathway and induce immune responses. Moreover, the released transition metal ions may bind to various enzymes as cofactors, thereby influencing their activities. Copper-based NMs (Cu^0^, Cu^+^ and Cu^2+^) possess great capacity for cancer therapy and antibacterial applications. Their dissolution or oxidation can result in the release of Cu^+^/Cu^2+^, which are believed to be essential dynamic regulators modulating mitochondrial respiration, autophagy and antioxidant defense in enzymatic and non-enzymatic manners. Iron oxide nanoparticles have been extensively used as effective imaging agents, immune modulators and drug carriers, and it has been shown that acidic conditions and enzymes in lysosomes are responsible for their biodegradations. Dissolved iron ions may then be incorporated into hemoglobins to maintain the regeneration of erythrocytes. Moreover, the bioavailability of TENMs as active biomolecules may induce beneficial consequences, especially under pathological conditions. Thus, when designing therapeutic nanoplatforms, more attention should be paid to their fate, biokinetic behavior and bioavailability *in vivo*, as well as the underlying mechanism of their medicinal efficacy.
